# Crystal structure of cobalt hydroxide carbonate Co_2_CO_3_(OH)_2_: density functional theory and X-ray diffraction investigation

**DOI:** 10.1107/S2052520617007983

**Published:** 2017-09-15

**Authors:** Jorge González-López, Jeremy K. Cockcroft, Ángeles Fernández-González, Amalia Jimenez, Ricardo Grau-Crespo

**Affiliations:** a Department of Geology, University of Oviedo, Calle Jesús Arias de Valasco s/n, Oviedo 33005, Spain; b Department of Chemistry, University College London, 20 Gordon Street, London WC1H 0AJ, UK; c Department of Chemistry, University of Reading, Whiteknights Campus, Reading RG6 6AD, England

**Keywords:** Co_2_CO_3_(OH)_2_, density functional theory, powder X-ray diffraction, rosasite, malachite

## Abstract

The cobalt carbonate hydroxide Co_2_CO_3_(OH)_2_ is a technologically important solid which is used as a precursor for the synthesis of cobalt oxides in a wide range of applications. It also has relevance as a potential immobilizer of the toxic element cobalt in the natural environment, but its detailed crystal structure is so far unknown. The structure of Co_2_CO_3_(OH)_2_ has now been investigated using density functional theory (DFT) simulations and powder X-ray diffraction (PXRD) measurements on samples synthesized *via* deposition from aqueous solution. Two possible monoclinic phases are considered, with closely related but symmetrically different crystal structures, based on those of the minerals malachite [Cu_2_CO_3_(OH)_2_] and rosasite [Cu_1.5_Zn_0.5_CO_3_(OH)_2_], as well as an orthorhombic phase that can be seen as a common parent structure for the two monoclinic phases, and a triclinic phase with the structure of the mineral kolwezite [Cu_1.34_Co_0.66_CO_3_(OH)_2_]. The DFT simulations predict that the rosasite-like and malachite-like phases are two different local minima of the potential energy landscape for Co_2_CO_3_(OH)_2_ and are practically degenerate in energy, while the orthorhombic and triclinic structures are unstable and experience barrierless transformations to the malachite phase upon relaxation. The best fit to the PXRD data is obtained using a rosasite model [monoclinic with space group *P*112_1_/*n* and cell parameters *a* = 3.1408 (4) Å, *b* = 12.2914 (17) Å, *c* = 9.3311 (16) Å and γ = 82.299 (16)°]. However, some features of the PXRD pattern are still not well accounted for by this refinement and the residual parameters are relatively poor. The relationship between the rosasite and malachite phases of Co_2_CO_3_(OH)_2_ is discussed and it is shown that they can be seen as polytypes. Based on the similar calculated stabilities of these two polytypes, it is speculated that some level of stacking disorder could account for the poor fit of the PXRD data. The possibility that Co_2_CO_3_(OH)_2_ could crystallize, under different growth conditions, as either rosasite or malachite, or even as a stacking-disordered phase intermediate between the two, requires further investigation.

## Abstract 2

The crystal structure of cobalt carbonate hydroxide Co_2_CO_3_(OH)_2_, a solid important in materials and environmental science, is investigated using density functional theory (DFT) simulations and powder X-ray diffraction (PXRD) measurements.

## 1. Introduction   

The solid structure of cobalt(II) carbonate hydroxide [Co_2_CO_3_(OH)_2_] is important for technological and environmental reasons. It is commonly used as a precursor in the synthesis of cobalt oxides (Li *et al.*, 2006 ▸, 2012 ▸; Xie *et al.*, 2010 ▸; Xu & Zeng, 2003 ▸), which have a wide range of technological applications as petroleum catalysts, magnetic materials, semiconductors, chemical gas sensors, solar collectors, lithium-ion batteries *etc*. (Ando *et al.*, 1997 ▸; Robert *et al.*, 2005 ▸; Tuti & Pepe, 2008 ▸; Wang *et al.*, 2008 ▸; Yuan *et al.*, 2003 ▸; Yang *et al.*, 2011 ▸). Co_2_CO_3_(OH)_2_ has also been proposed as a potential immobilizer of cobalt in the natural environment (Katsikopoulos *et al.*, 2008 ▸). Cobalt is considered as a possible carcinogen by the International Agency for Research on Cancer (IARC, 1991 ▸). Moreover, some of its isotopes (^58^Co and ^60^Co) are radioactive, which makes them useful in nuclear applications but also implies risks to human health. Although cobalt appears only as a trace element in the Earth’s crust (Smith & Carson, 1981 ▸), it can be found more abundantly in soils and groundwater as a consequence of the extraction process of Co-bearing minerals, and also as waste derived from industrial activities, *e.g.* construction (alloy steel), use of cobalt-containing fertilisers, manufacture of pigments, batteries *etc*. (ATSDR, 2004 ▸). Previous research has considered possible routes for cobalt immobilization (*via* precipitation and/or interaction) by carbonate-containing materials, in particular calcite CaCO_3_ (Katsikopoulos *et al.*, 2008 ▸; Wada *et al.*, 1995 ▸; Braybrook *et al.*, 2002 ▸). However, no clear incorporation in calcite has been observed. In fact, a theoretical study of the thermodynamic properties of Ca_1−*x*_Co*_x_*CO_3_ solid solutions concluded that no significant amount of cobalt can be expected to incorporate substitutionally in the calcite structure under ambient conditions (González-López *et al.*, 2014 ▸). Since cobalt immobilization in aqueous environments *via* calcite precipitation seems to be difficult to achieve, there is interest in investigating other phases that could immobilize cobalt. The first substance precipitated from cobalt and carbonate ions in aqueous solution at ambient temperature is known to be an amorphous phase (Barber *et al.*, 1975 ▸). Katsikopoulos *et al.* (2008 ▸) reported that this amorphous substance corresponds to a hydrated cobalt carbonate. These authors showed that the precipitation from Co^2+^ and CO_3_^2−^ at room temperature from aqueous solution leads to a transformation from the amorphous carbonate to a carbonate phase with better crystallinity, through aging in the same aqueous solution from where it has been precipitated. Thus, amorphous and crystalline cobalt hydroxide carbonate phases are likely to exist in areas of the Earth’s crust where Co is anomalously present in contact with ground and fresh waters (*e.g.* mining, waste disposal sites *etc*.), and these phases might play an important role in cobalt immobilization in the natural environment.

The detailed crystal structure of Co_2_CO_3_(OH)_2_ is so far unknown. A preliminary powder X-ray diffraction (PXRD) study by Wang *et al.* (2009 ▸) suggested a malachite-type monoclinic structure with space group *P*12_1_/*a*1 and *a* = 9.448 Å, *b* = 12.186 Å, *c* = 3.188 Å and β = 91.879°, but the atomic positions were not refined. In a short conference report later (Wang *et al.*, 2010 ▸), these authors described a refinement attempt, but the reported positions are unlikely to be correct (there are no defined CO_3_ units nor CoO_6_ octahedra) and are not comparable with those in the malachite structure. On the other hand, some of us have recently reported the PXRD characterization of synthetic Co_2_CO_3_(OH)_2_ and indexed the structure as a rosasite-like monoclinic structure with space group *P*12_1_/*a*1 and *a* = 12.886 Å, *b* = 9.346 Å, *c* = 3.156 Å and β = 110.358°, but we did not attempt to refine the atomic positions either, due to the low crystallinity of the samples (González-López *et al.*, 2016 ▸).

As will be seen in more detail below, the malachite-like and rosasite-like structures, while closely related and expressed in the same space group, are not isotypic. The relationship between them has been discussed before by Girgsdies & Behrens (2012 ▸), where an orthorhombic structure with space group *Pbam* was also proposed as a common hypothetical parent structure (aristotype). Interestingly, some authors have assigned the Co_2_CO_3_(OH)_2_ structure to the orthorhombic crystal system, although again no atomic positions were reported (Yang *et al.*, 2011 ▸; Xing *et al.*, 2008 ▸). Finally, there is also a triclinic structure associated with the *M*CO_3_(OH)_2_ stoichiometry, which is that of the mineral kolwezite [Cu_1.34_Co_0.66_CO_3_(OH)_2_] where the three cell angles are close to 90° (Deliens & Piret, 1980 ▸).

The objective of the present work was to elucidate the crystal structure of Co_2_CO_3_(OH)_2_ using a combination of density functional theory (DFT) calculations and PXRD measurements on hydro­thermally synthesized samples. We have investigated the thermodynamic stability of Co_2_(OH)_2_CO_3_ in each of the two monoclinic phases (rosasite and malachite), in the orthorhombic aristotype structure, and in the triclinic kolwezite structure. We then used the DFT models to aid the interpretation of the PXRD patterns.

## 2. Methodology   

### 2.1. Density functional theory calculations   

The equilibrium geometries and energies of different possible phases of Co_2_CO_3_(OH)_2_ were calculated using DFT simulations, as implemented in the *VASP* code (Kresse & Furthmüller, 1996*a* ▸,*b* ▸). We employed the generalized gradient approximation (GGA) with the PBE exchange correlation functional (Perdew *et al.*, 1996 ▸). In order to improve the description of the highly localized Co 3*d* orbitals, we employed the so-called GGA+U correction scheme, where we used a Hubbard parameter *U*_eff_ = 6.1 eV, which is the value found for Co 3*d* by Wdowik & Parlinski (2007 ▸), to reproduce the experimental band gap of cobalt(II) oxide (CoO). All calculations were performed allowing spin polarization, as the Co^II^ cations formally have the electronic configuration 3*d* ^7^. We tested both low-spin and high-spin configurations with different magnetic orderings, and found that the Co^II^ ions always prefer to be in high-spin configurations (three unpaired electrons or *S* = 3/2) with the magnetic moments being weakly coupled (energy differences between ferromagnetic and antiferromagnetic configurations will be discussed below). The interaction between the valence electrons and the core was described using the projected augmented wave (PAW) method (Blöchl, 1994 ▸) in the implementation of Kresse & Joubert (1999 ▸). The core levels up to 3*s* in Ca, 3*p* in Co, and 1*s* in C and in O were kept frozen in their atomic reference states. The number of plane waves in *VASP* is controlled by a cutoff energy, in our case 520 eV, which is 30% higher than the standard value for the PAW potentials employed. For reciprocal-space integrations we used a Γ-centred *k*-point mesh of 8, 3 and 2 divisions along the short, medium and long axes of the structures, respectively (the corresponding lengths are similar for the malachite and rosasite structures). We checked that these settings of cutoff energy and *k*-point grids lead to total energies converging within 1 meV per formula unit (the convergence in relative energies is likely to be even better). Each structure was fully relaxed (both cell parameters and ion coordinates) to the equilibrium geometry using a conjugate gradients algorithm until the forces on the atoms were all less than 0.01 eV Å^−1^.

### 2.2. Sample preparation and electron microscopy imaging   

We synthesized the cobalt hydroxide carbonate using a hydro­thermal method to ensure complete crystallization. A 0.05 *M* aqueous solution of CoCl_2_·6H_2_O was mixed with the same volume of a 0.05 *M* aqueous solution of Na_2_CO_3_. The mixing was done in a jacketed glass reactor equipped with an entry for a thermocouple in order to regulate the temperature. The final solution was kept at 338 K with constant stirring for 6 d. After the reaction time, the aqueous solution was cooled to room temperature and then filtered using a 0.45 Millipore paper filter. The solid was dried at room temperature and then powdered in an agate mortar. Although sample preparation at higher temperatures could in principle lead to better crystallinity, this is complicated by the formation of Co_3_O_4_. For example, a synthesis attempt increasing the temperature from 338 to 403 K for 1 d failed to produce cobalt hydroxide carbonate and led instead to Co_3_O_4_, as confirmed by Raman analysis.

Scanning electron microscopy (SEM) and transmission electron microscopy (TEM) images were taken in a JEOL 6610LV and a JEOL JEM-2100F microscope, respectively. Each instrument was equipped with an energy dispersive X-ray microanalysis system supplied with a silicon drift detector.

### 2.3. X-ray diffraction measurements   

Powder X-ray diffraction measurements were made using a Stoe STADI-P powder diffractometer equipped with an Mo X-ray anode (set to 50 kV, 40 mA), a Ge(111) monochromator providing Mo *K*α_1_ radiation (nominal wavelength λ = 0.7093 Å), a reduced axial-divergence collimator and a Mythen 1K detector. Mo X-ray radiation was used instead of the more common Cu X-ray radiation to avoid fluorescence from Co in the sample. The sample was mounted in a 0.5 mm X-ray glass capillary. Diffraction patterns were measured from 1 to 50° in 2θ with a detector step of 0.2° at 120 seconds per step with the data binned in 0.015° in 2θ. This scan was repeated five times to improve the statistical quality of the diffraction patterns and the data totalled.

## 3. Results and discussion   

Our DFT calculations started from structures based on experimental data on rosasite (Perchiazzi, 2006 ▸), malachite (Süsse, 1967 ▸) and kolwezite (Deliens & Piret, 1980 ▸) minerals, substituting the metal atoms in the original minerals by cobalt. We also used an orthorhombic structure based on the parameters given by Girgsdies & Behrens (2012 ▸) as a starting point. Upon relaxation, both the kolwezite and orthorhombic structures converged to the same structure as malachite, while the rosasite converged to a distinct structure. In the language of potential energy landscapes, we can say that the malachite and rosasite structures are two different local minima, whereas the kolwezite and orthorhombic structures are both within the basin of the malachite minimum. The distinctiveness of the malachite and rosasite structures is clear from the observation that in the former the monoclinic angle is between the short and medium cell vectors, while in the latter it is between the short and long cell vectors. In what follows we deal only with the malachite and rosasite structures, as the other two are unstable.

**Figure 1:**
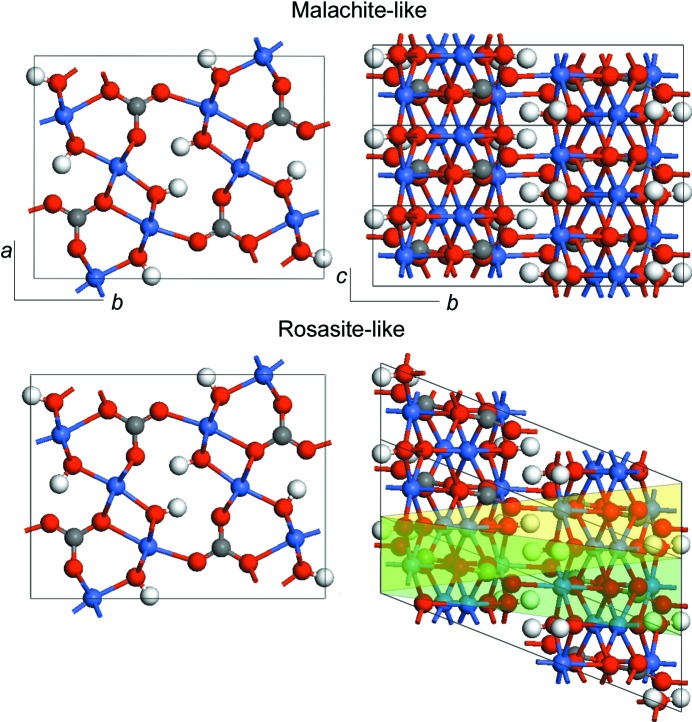
Malachite-like (top) and rosasite-like (bottom) crystal structures of Co_2_CO_3_(OH)_2_ as obtained from DFT calculations. The rosasite-like structure is displayed with the atomic positions shifted in a way that maximizes the coincidence with the malachite structure and does not follow the values listed in Table 2 ▸. Colour shading is used to represent alternative cells with different values of the monoclinic angle. The green-shaded cell was used for the DFT calculations. Colour code: Co blue, C grey, O red and H white.

In order to achieve a fair comparison between the energies of the malachite and rosasite structures, we chose the crystallographic axes for the latter in a way that is different from the setting used originally by Perchiazzi (2006 ▸) for the rosasite mineral [Cu_1.20_Zn_0.80_CO_3_(OH)_2_] and by us in our previous work on Co_2_CO_3_(OH)_2_ (González-López *et al.*, 2016 ▸). As can be seen in Fig. 1 ▸, the monoclinic angle in the rosasite structure can be chosen in different ways, depending on the unit-cell definition, and we have simply used the one that gives a value closer to 90° upon relaxation (the green cell in the figure), since that leads to maximum similarity with the malachite structure.

We have assessed the relative stabilities of the rosasite- and malachite-like structures in ferromagnetic and antiferromagnetic configurations for each structure. The Co cations are directly connected by oxygen anions along both the *a* and *c* directions (with reference to the malachite unit-cell axes), allowing for superexchange coupling, but are separated by the carbonate species along the *b* direction, leading to an effectively two-dimensional (even if geometrically not flat) network of coupled magnetic centres. Due to the periodicity of the simulation cell, we can enforce antiferromagnetic alternation of the magnetic moments along the *a* direction but not along the *c* direction (in which neighbouring ions are periodic images of one another). Creating a supercell along the *c* direction would allow us to explore different antiferromagnetic configurations, but we have observed that the relative energies of the malachite-like and rosasite-like structures are almost independent of the magnetic configurations, so the consideration of larger supercells is not necessary for the purpose of this study. Table 1 ▸ shows that, for both structures, the antiferromagnetic configuration is more stable by ∼17 meV per formula unit. The rosasite-like and malachite-like structures are practically degenerate in energy, with a calculated energy difference (∼0.05 meV per formula unit) that is too small to be meaningful, considering the general precision of DFT simulations.

**Figure 2:**
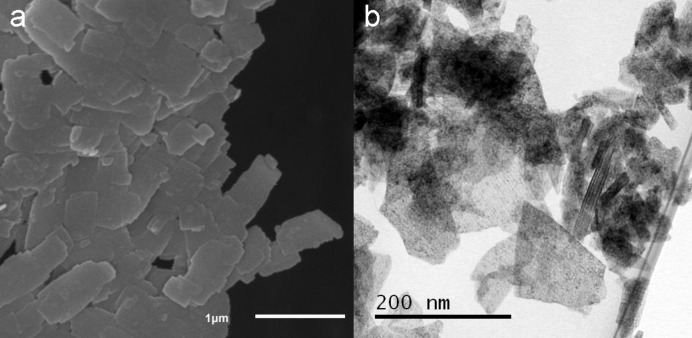
(*a*) SEM and (*b*) TEM images of Co_2_CO_3_(OH)_2_.

We therefore turn to experimental measurements in order to compare (refined) Rietveld models based on the DFT structures with the PXRD patterns. Our cobalt hydroxide carbonate sample obtained at 338 K is shown in the electron microscopy images in Fig. 2 ▸. Both the SEM image (Fig. 2 ▸ *a*) and the TEM image (Fig. 2 ▸ *b*) show well formed nanocrystals which exhibit a clear ‘plate’ morphology, in agreement with previous reports (Wang *et al.*, 2009 ▸; Zhang *et al.*, 2013 ▸).

**Figure 3:**
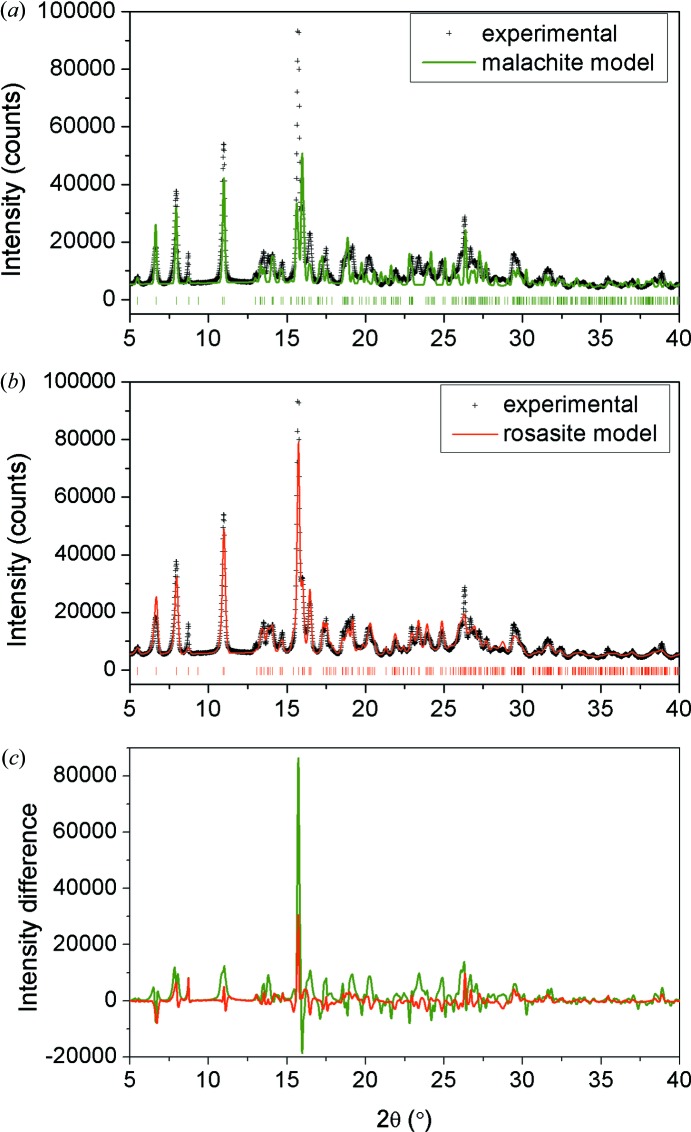
Experimental X-ray diffraction pattern (+ symbols) compared with (*a*) malachite-like and (*b*) rosasite-like (green and red lines, respectively) Rietveld refinement curves (atomic positions fixed to DFT values). (*c*) Difference between experimental and refined intensities for both models.

Fig. 3 ▸ shows the experimental PXRD diffraction pattern of the sample. Using the DFT-generated malachite and rosasite structures within the Rietveld refinement program *Rietica* (version 1.77; Hunter, 1998 ▸), peak position and shape parameters were refined by least-squares fits to the PXRD data with atomic coordinates kept fixed to the DFT values. The calculated pattern for the malachite model is shown in green in Fig. 3 ▸(*a*) and that for the rosasite model in red in Fig. 3 ▸(*b*). Intensity difference plots for both models are shown in Fig. 3 ▸(*c*). The results show that the rosasite-type model gives the best fit to the experimental diffraction data (*R*_wp_ = 12.9%, compared with 32.6% for the fit with the malachite model). However, there are still systematic differences in peak intensities between the PXRD data and the rosasite-based Rietveld model, which cannot be resolved by refinement and therefore can be ascribed to the model itself. The refinement of individual atomic coordinates does not result in a significant improvement in the fit to the PXRD data: the *R*_wp_ can be only slightly reduced by full refinement (from 12.9% to 12.6%), but the resulting coordinates are no more reliable than the DFT ones, since the refinement simply attempts to correct for the peak intensities that cannot be fully described by the rosasite model. Tables 2 ▸ and 3 ▸ show the DFT-calculated and Rietveld-refined cell parameters, as well as the atomic coordinates from DFT, for the rosasite and malachite models, respectively.

It is interesting to note here that Perchiazzi & Merlino (2006 ▸), in their study of the related compound Mg_2_CO_3_(OH)_2_, discussed its possible non-stoichiometry in the form of metal cation vacancies. We have also considered here the refinement of the Co_2_CO_3_(OH)_2_ structure varying the site occupancies for both Co1 and Co2 positions in the rosasite structure. For Co2, the site occupation number stays at around 100% and the *R* factor does not improve. Interestingly, for Co1 the occupancy drops to around 87% with a 1% improvement in *R*_wp_. However, the *R*_wp_ is still relatively high at 11.9% because the most intense peak is still poorly fitted by the model. We therefore believe that this result, although interesting enough to be reported, should not be taken as a strong suggestion of the presence of Co vacancies in this cobalt hydroxide carbonate. Given the limitations of the rosasite model, anything that slightly improves the intensity of the most intense peak will reduce *R*_wp_, so the fractional occupancy may simply be an artefact of the fit. The potential presence of cation vacancies in this compound requires further investigation in future work.

**Figure 4:**
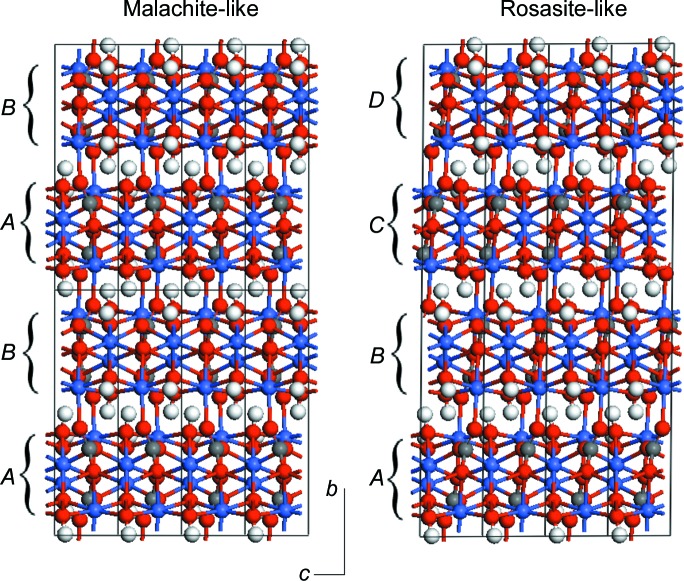
Malachite-like and rosasite-like structures of Co_2_CO_3_(OH)_2_ seen as two different stacking sequences of the same two-dimensional motif. The rosasite-like structure is shown using a redefined supercell lattice for better comparison with the malachite-like structure. Colour code as in Fig. 1 ▸.

Finally, we discuss possible reasons as to why neither the rosasite nor the malachite model gives a completely satisfactory fitting of the PXRD data. A possible explanation, consistent with the small DFT energy difference between the two structures, is that both phases coexist in the sample. However, a two-phase Rietveld refinement does not significantly improve the fit (as measured by *R*_wp_ and by visual appearance). The refined scale factors from the two-phase model show that the amount of malachite phase present, if any, is insignificant. A closer look at both structures offers a more interesting possible explanation. Fig. 4 ▸ shows the two structures in a plane perpendicular to the (malachite) *a* axis (the rosasite axes have been redefined again here to show the analogy with malachite). They can be seen as structures made up of identical layers but with different stacking sequences. The relative lateral shifts from one layer to the next are always the same in each structure, involving a ¼ shift along the malachite *c* axis. But while in malachite consecutive shifts are in opposite directions, leading to an *ABAB* sequence, in rosasite the shifts are always in the same direction, leading to an *ABCD* sequence. Therefore the two structures can be considered as polytypes.

The fact that not only the layer structure but also the local geometry of the interface are the same for both structures explains their very similar energies: the only difference between the two structures is in the interaction between next-nearest layers. Our results therefore suggest that actual samples might exhibit stacking disorder, with random relative directions of consecutive shifts, instead of the two well ordered shift patterns represented by the malachite- and rosasite-like structures. This interesting possibility requires further theoretical and experimental investigation. For the moment, the rosasite-like model reported here is the best available model for the Co_2_CO_3_(OH)_3_ structure.

## Supplementary Material

Crystal structure: contains datablock(s) global, I. DOI: 10.1107/S2052520617007983/wf5134sup1.cif

Rietveld powder data: contains datablock(s) I. DOI: 10.1107/S2052520617007983/wf5134Isup2.rtv

CCDC reference: 1553059

## Figures and Tables

**Table 1:** Relative DFT energies for the malachite-like and rosasite-like structures of Co_2_CO_3_(OH)_2_ in the ferromagnetic (FM) and antiferromagnetic (AFM) configurations described in the main text

	*E* (meV per formula unit)	
Structure	AFM	FM
Malachite	0	16.92
Rosasite	0.04	16.98

**Table:** Rietveld-refined values of cell parameters are given within square brackets.

Space group	*P*112_1_/*n*
*a* (Å)	3.174 [3.1408 (4)]
*b* (Å)	12.374 [12.2914 (18)]
*c* (Å)	9.413 [9.3311 (16)]
γ (°)	82.82 [82.299 (16)]

**Table:** 

Coordinates	*x*	*y*	*z*
Co1	0.77660	0.71075	0.49778
Co2	0.18314	0.89784	0.26841
C	0.38881	0.64742	0.22817
O1	0.30694	0.64639	0.36513
O2	0.28751	0.73926	0.15774
O3	0.57404	0.56386	0.16515
O4	0.70019	0.85789	0.40510
O5	0.67413	0.91997	0.12379
H1	0.31773	0.00498	0.90820
H2	0.27813	0.09536	0.51076

**Table:** Rietveld-refined values of cell parameters are given within square brackets; however, note that the quality of the fit with this model is poor – see text.

Space group	*P*12_1_/*a*1
*a* (Å)	9.425 [9.307 (2)]
*b* (Å)	12.261 [12.224 (2)]
*c* (Å)	3.174 [3.1346 (7)]
β (°)	91.12 [90.486 (16)]

**Table:** 

Coordinates	*x*	*y*	*z*
Co1	0.00262	0.28894	0.86602
Co2	0.73213	0.39792	0.3694
C	0.77217	0.14755	0.45237
O1	0.63493	0.1467	0.36907
O2	0.84296	0.2389	0.38999
O3	0.83503	0.06414	0.60326
O4	0.59572	0.3581	0.86532
O5	0.87648	0.42001	0.8779
H1	0.51153	0.40484	0.81598
H2	0.90773	0.49536	0.83501
